# Parental mental disorders in patients with comorbid schizophrenia and obsessive–compulsive disorder: a nationwide family-link study

**DOI:** 10.1007/s00787-024-02480-0

**Published:** 2024-05-30

**Authors:** Tien-Wei Hsu, Shih-Jen Tsai, Ya-Mei Bai, Chih-Ming Cheng, Tung-Ping Su, Tzeng-Ji Chen, Chih-Sung Liang, Mu-Hong Chen

**Affiliations:** 1 Department of Psychiatry, E-DA Dachang Hospital, I-Shou University, Kaohsiung, Taiwan; 2 Graduate Institute of Clinical Medicine, College of Medicine, Kaohsiung Medical University, Kaohsiung, Taiwan; 3 Department of Psychiatry, Taipei Veterans General Hospital, Beitou District, No. 201, Sec. 2, Shihpai Road, Taipei, 11217 Taiwan; 4 Department of Psychiatry, College of Medicine, National Yang Ming Chiao Tung University, Taipei, Taiwan; 5 Department of Psychiatry, Cheng Hsin Hospital, Taipei, Taiwan; 6 Department of Family Medicine, Taipei Veterans General Hospital, Taipei, Taiwan; 7 Institute of Hospital and Health Care Administration, National Yang Ming Chiao Tung University, Taipei, Taiwan; 8 Department of Family Medicine, Hsinchu Branch, Taipei Veterans General Hospital, Hsinchu, Taiwan; 9 Department of Psychiatry, Beitou Branch, Tri-Service General Hospital, No. 60, Xinmin Road, Beitou District, Taipei, 11217 Taiwan; 10 Department of Psychiatry, National Defense Medical Center, Taipei, Taiwan

**Keywords:** Schizophrenia, Obsessive–compulsive disorder, Bipolar disorder, Depressive disorder, Substance use disorder

## Abstract

Schizophrenia is highly comorbid with obsessive–compulsive disorder (OCD); both conditions share numerous pathophysiological etiologies. We, thus, examined the risk of mental disorders in the parents of probands with schizophrenia, OCD, or both conditions. Between 2001 and 2011, we enrolled a nationwide cohort of 69,813 patients with schizophrenia, OCD, or both. The control cohort included 698,130 individuals matched for demographics. Poisson regression models were employed to examine the risk of six mental disorders in their parents, including schizophrenia, bipolar disorder, depressive disorder, OCD, alcohol use disorder, and substance use disorder. We stratified patients into schizophrenia-only, OCD-only, and dual-diagnosis groups, and the dual-diagnosis group was further divided into schizophrenia-first, OCD-first, and simultaneously diagnosed groups. Compared with controls, the schizophrenia, OCD, and dual-diagnosis groups had higher risks for the six mental disorders in their parents (range of odds ratio [OR] 1.50–7.83). The sub-analysis of the dual-diagnosis group showed that the schizophrenia-first, OCD-first, and simultaneously diagnosed groups had higher odds for schizophrenia, bipolar disorder, depressive disorder, and OCD (range of OR 1.64–6.45) in their parents than the control group; the simultaneously diagnosed and OCD-first diagnosed groups had a higher odds of parental substance use disorder, while the schizophrenia-first diagnosed group had a higher odds of parental alcohol use disorder. The interrelationship between OCD and schizophrenia is linked to bipolar disorder, depressive disorder, alcohol use disorder, and substance use disorder. The results have implications for mental health policy and future research.

**Supplementary Information:**

The online version contains supplementary material available at 10.1007/s00787-024-02480-0.

## Introduction

The high comorbidity of schizophrenia and obsessive–compulsive disorder (OCD) has been widely researched [1]. A meta-analysis published in 2014 reported that the prevalence of OCD in patients with schizophrenia was 12.3% [2], while it was 2–3% in the general population [3]. Patients with comorbid schizophrenia and OCD (dual diagnosis) had a different pattern of comorbidity from those with schizophrenia only. Patients with dual diagnosis had higher comorbidity with body dysmorphic disorder, eating disorders, and tic disorders than the general population; however, this was not true for patients with major depressive disorder and substance use disorder [4]. In addition, patients with dual diagnosis also had more severe positive and negative symptoms, higher incidence of suicidality and neurological soft signs, poorer neurocognitive and global function, and lower quality of life than those with schizophrenia only [1, 5].

The high comorbidity of schizophrenia and OCD may be explained by substantial overlapping pathophysiologies. Neuroimaging studies have reported functional and structural similarities over the neuroanatomical regions in these two disorders, including in the caudate nucleus, the orbitofrontal cortex, the anterior cingulate gyrus, and the mediodorsal thalamic nucleus [6, 7]. Genetic studies on schizophrenia and OCD have indicated shared genes that are involved in the dopaminergic system, serotonergic receptors, voltage-gated calcium ion channels, and regulation of the hippo signaling pathway [8]. A large body of data from a of genomic copy number variation study has also reported that these two disorders had several common mutations, such as 15q11-q13 duplication and 16p13.11 duplication [9]. Importantly, atypical antipsychotics (e.g., clozapine) were considered inducers of obsessive–compulsive symptom development in patients with schizophrenia, and the mechanism has been associated with sequence variations in the glutamate transporter gene SLC1A1 [10, 11]. Obsessive–compulsive symptoms are also the prodromal symptoms of schizophrenia [12]. When considering these findings together, it is found that the sequence of occurrence of OCD and schizophrenia might imply different etiologies for patients with comorbid schizophrenia and OCD.

Family-based studies have supported the link between OCD and schizophrenia. A large longitudinal and multi-generational study showed that parents of probands with OCD had a 2.2-fold higher odds of schizophrenia compared with parents of probands without OCD [13]. Another longitudinal study found that parental OCD was associated with a 4.86-fold higher risk of schizophrenia in the offspring compared with that in the offspring of parents without OCD [14]. Importantly, the relatives of patients with schizophrenia had high risk of other mental disorders, such as bipolar disorder and depressive disorder [15], while anxiety disorders, somatoform disorders, and mood disorders were common in the relatives of patients with OCD [16]. To date, few studies have examined the odds of mental disorders in the parents of patients with both OCD and schizophrenia. Only a small familial aggregation study (n = 100) reported that the first-degree relatives of probands with dual diagnosis had higher risks for schizo-obsessive disorder and obsessive–compulsive personality disorder, but not for major depressive disorder, bipolar disorder, and substance use disorders, compared with the control probands [4].

In a clinical scenario, we would investigate whether the parents of a patient newly diagnosed with OCD or schizophrenia have a history of mental disorders. We would also be curious about whether there are differences in the odds of mental disorders among the parents of probands with schizophrenia only, OCD only, or with dual diagnoses. To answer these questions, we conducted this longitudinal family-link study, linking the diagnosis of mental disorders from the schizophrenia/OCD probands' generation to their parents. We also investigated the association of the sequence of the two disorders (simultaneously vs. schizophrenia first vs. OCD first) with the odds of mental disorders in their parents.

## Methods

### Data acquisition

Taiwan's National Health Insurance, a mandatory universal health insurance program, offers comprehensive medical care coverage to all Taiwanese residents (more than 23 million people). The Taiwan National Health Insurance Research Database (NHIRD) is audited and released by the Taiwan National Health Research Institute for scientific studies [17, 18]. Information on insured individuals is included in the NHIRD, such as demographic data, clinical visit dates, and disease diagnoses. The insurance claim information of the individuals is anonymized to maintain privacy. All of the information used in the present study was linked using each resident’s unique personal identification number. Subsequently, following the method of Chen et al. and Cheng et al., family kinships in the NHIRD were used for genealogy reconstruction [19, 20]. The registry of beneficiaries contains the identifiers of the relationships between the insured person (who paid the insurance fee) and his/her dependents. Only blood relatives or spouses were qualified to be dependents of the insured patients. With unique personal identifiers, parent–offspring relationships and spouses can be identified directly. The diagnostic codes used were based on the International Classification of Diseases, 9th Revision, Clinical Modification (ICD-9-CM). The NHIRD has been used extensively in many Taiwanese epidemiologic studies [19–23]. This study protocol was reviewed and accepted by the Institutional Review Board of Taipei Veterans General Hospital.

### Inclusion criteria of probands with OCD and schizophrenia and the controls

Between January 1 2001, and December 31 2011, we enrolled adolescents aged between 12 and 17 years and adults aged between 18 and 64 years with a diagnosis of schizophrenia (ICD-9-CM code: 295) or OCD (ICD-9-CM code: 300.3) or with dual diagnosis of schizophrenia and OCD as the study cohort.

If there are changes or additions to the diagnosis during the following period, the latest diagnosis given by board-certified psychiatrist twice will take precedence. For each case, we randomly selected a 1:10 matched control based on age (± 1 year), sex, income, and residence after eliminating those individuals with any major psychiatric disorder (ICD-9-CM codes: 291, 292, 295, 296, 300.3, 300.4, 303, 304, 305 except 305.1, and 311) anytime in the database (supplementary material). During the same study period, we identified six mental disorders in the probands’ and controls’ parents, including schizophrenia, bipolar disorder, depressive disorder, OCD, alcohol use disorder, and substance use disorder. To ensure the diagnostic validity, the diagnoses of the six mental disorders were required to be given by board-certified psychiatrists at least twice. Income level (levels 1–3 per month: ≤ 19,100 NTD (New Taiwanese Dollars), 19,001 ~ 42,000 NTD, and > 42,000 NTD) and urbanization level of residence (levels 1–5, most to least urbanized) were assessed as the proxy for healthcare availability in Taiwan [24].

### Statistical analysis

Chi-square statistics and F-tests were used to compare categorical and continuous variables, respectively, between the four groups of schizophrenia only, OCD only, combined, and control groups. Some offspring were from the same families, resulting in a clustered study sample [25]. After adjustment for demographic data (age, sex, residence, and income), we used Poisson regression models with a robust error variance to calculate the odds ratios (ORs) with 95% confidence intervals (CIs) for the odds of parental mental disorders in probands with schizophrenia only, OCD only, or dual diagnosis compared with those in controls. Sex-stratified subanalysis was performed to examine the odds of the six mental disorders in male probands and female probands compared with those in the matched controls. We also investigated the impact of the temporal relationship of first onset of OCD vs schizophrenia in the probands on the odds of the six mental disorders in their parents. The dual-diagnosis group was divided into three subgroups: (1) first onset of OCD, (2) first onset of schizophrenia, and (3) simultaneous onset of OCD and schizophrenia. The simultaneous onset of OCD and schizophrenia was defined as < 365 days of the interval between schizophrenia and OCD diagnosis times. Finally, the entire sample was divided to adolescent (< 18 years) and adult (≥ 18 years) samples based on the age of the first diagnosis for the sensitivity analyses. For example, if a boy who was diagnosed with OCD at age 15 years and subsequently was diagnosed with schizophrenia at age 21 years, he was classified in the adolescent sample. SAS 9.2 (SAS Institute, Cary, NC, USA) was used for all statistical analyses. All tests were two-tailed, and p < 0.05 was considered statistically significant.

## Results

We enrolled 48,201 probands with schizophrenia, 17,809 probands with OCD, 3,803 probands with dual diagnosis, and 698,130 matched controls without any major psychiatric disorder (Table 1). The mean age was 35.5 (standard deviation, SD = 10.53) years in the schizophrenia-only group, 30.69 (9.92) years in the OCD-only group, 31.63 (8.22) years in the dual-diagnosis group and 34.06 (10.49) years in the control group (group difference: p < 0.001). Males accounted for 56.2% in the schizophrenia-only group, 57.1% in the OCD-only group, 63.1% in the dual-diagnosis group, and 56.8% in the control group (group difference: p < 0.001). The age at schizophrenia diagnosis was 27.81 (9.46) years in the schizophrenia-only group and 24.17 (7.48) years in the dual-diagnosis group. The age at OCD diagnosis was 25.21 (9.39) years in the OCD-only group and 25.03 (8.04) years in the dual-diagnosis group. Among those in the dual-diagnosis group, 43.8% were simultaneously diagnosed with OCD and schizophrenia, 36.5% were diagnosed with schizophrenia first, and 19.7% were diagnosed with OCD first. The prevalence of the six mental disorders varied among the four groups, and group differences in the prevalence of the six mental disorders were statistically significant (all p < 0.001). The rate of diagnosis of schizophrenia was highest in the schizophrenia-only group (3.9%); that of bipolar disorder (2.6%) and depressive disorder (8.0%) were highest in the dual-diagnosis group; and that of alcohol use disorder (1.7%), substance use disorder (1.5%), and OCD (1.5%) were highest in the OCD-only group (Table 1). The level of urbanization and the income were significantly different among the four groups (both p < 0.001).

**Table 1:** Demographic characteristics and prevalence of parental mental disorders between patients and controls

	A. Schizophrenia only group (n = 48,201)	B. OCD only group (n = 17,809)	C. Dual diagnosis group (n = 3803)	D. Control group (n = 698,130)	p-value	Post-hoc
Age in 2011 (years, SD)	35.50 (10.53)	30.69 (9.92)	31.63 (8.22)	34.06 (10.49)	< 0.001	A > D > C > B
Birth year (n, %)					< 0.001	
< 1970	11,681 (24.2)	2067 (11.6)	418 (11.0)	141,682 (20.3)		
1970–1975	7183 (14.9)	1765 (9.9)	492 (12.9)	94,486 (13.5)		
1976–1980	11,324 (23.5)	3679 (20.7)	943 (24.8)	159,592 (22.9)		
1981–1985	9868 (20.5)	4341 (24.4)	1011 (26.6)	152,132 (21.8)		
1986–1990	5528 (11.5)	3420 (19.2)	663 (17.4)	95,824 (13.7)		
1991–1995	2334 (4.8)	2070 (11.6)	254 (6.7)	46,728 (6.7)		
≥ 1996	283 (0.6)	467 (2.6)	22 (0.6)	7685 (1.1)		
Age at offspring birth of parents (years, SD)						
Father	31.20 (6.67)	30.65 (5.72)	31.27 (6.06)	29.91 (5.82)	< 0.001	A ~ C > B > D
Mother	26.58 (4.96)	27.15 (4.69)	27.23 (4.54)	26.26 (4.89)	< 0.001	B ~ C > A > D
Male (n, %)	27,097 (56.2)	10,157 (57.1)	2401 (63.1)	396,730 (56.8)	< 0.001	
Age at schizophrenia diagnosis (years, SD)	27.81 (9.46)		24.17 (7.48)			
Age at OCD diagnosis (years, SD)		25.21 (9.39)	25.03 (8.04)			
Sequence of diagnoses (n, %)						
Simultaneously			1667 (43.8)			
Schizophrenia first			1388 (36.5)			
OCD first			748 (19.7)			
Diagnoses of parental mental disorders (n, %)						
Schizophrenia	1895 (3.9)	186 (1.0)	112 (2.9)	4979 (0.7)	< 0.001	A > C > B > D
Bipolar disorder	1091 (2.3)	331 (1.9)	100 (2.6)	5122 (0.7)	< 0.001	C > A > B > D
Depressive disorder	2807 (5.8)	1352 (7.6)	304 (8.0)	21,496 (3.1)	< 0.001	B ~ C > A > D
Alcohol use disorder	767 (1.6)	298 (1.7)	61 (1.6)	7010 (1.0)	< 0.001	A ~ B ~ C > D
Substance use disorder	569 (1.2)	262 (1.5)	53 (1.4)	5291 (0.8)	< 0.001	B ~ C > A > D
OCD	152 (0.3)	261 (1.5)	41 (1.1)	1211 (0.2)	< 0.001	B > C > A > D
Level of urbanization (n, %)					< 0.001	
1 (most urbanized)	15,779 (21.9)	5779 (27.3)	1261 (23.2)	163,010 (23.3)		
2	6778 (32.7)	2064 (32.4)	458 (33.2)	228,190 (32.7)		
3	5620 (14.1)	1580 (11.6)	410 (12.0)	93,000 (13.3)		
4	9470 (11.7)	3522 (8.9)	791 (10.8)	76,100 (10.9)		
5 (most rural)	15,779 (19.6)	5779 (19.8)	1261 (20.8)	137,830 (19.8)		
Income-related insured amount (n, %)					< 0.001	
≤ 19,100 NTD/month	17,709 (36.7)	3254 (18.3)	1177 (30.9)	219,158 (31.4)		
19,001 ~ 42,000 NTD/month	18,212 (37.8)	5706 (32.0)	1289 (33.9)	248,225 (35.6)		
> 42,000 NTD/month	12,280 (25.5)	8849 (49.7)	1337 (35.2)	230,747 (33.0)		

*OCD* obsessive compulsive disorder, *SD* standard deviation, *NTD* new Taiwan dollar

After adjustments for demographic variables, all the three groups (schizophrenia only, OCD only, and dual diagnosis) had associations with increased odds of the six mental disorders than the control group (reported as OR with 95% Cis; OR range: 1.50–7.83), with the highest odds of OCD in the parents of proband with OCD only (7.83, 6.83–9.97) (Table 2). Compared with the controls, the probands with schizophrenia had the highest odds for schizophrenia (5.61, 5.31–5.92) in their parents, followed by bipolar disorder (3.20, 2.99–3.41) and depressive disorder (1.96, 1.88–2.04); the probands with OCD had the highest odds for OCD (7.83, 6.83–8.97) in their parents, followed by depressive disorder (2.56, 2.42–2.71) and bipolar disorder (2.45, 2.19–2.74); the probands with dual diagnosis had the highest odds for OCD (6.01, 4.39–8.22) in their parents, followed by schizophrenia (4.06, 3.35–4.91) and bipolar disorder (3.48, 2.85–4.26). The odds for alcohol use disorder and substance use disorder were similar for the three groups. When stratified by sex, most of the analyses remained significant in either the male or the female probands, except for the parental odds of substance use disorder and alcohol use disorder in female probands with dual diagnosis (Table 2). When stratified by adolescence and adult, except for the parental odds of alcohol use disorder in adult probands, most of the analyses were significant in both groups (Table 2).

**Table 2:** Risks of parental mental disorders between schizophrenia only, OCD only, dual-diagnosis, and control groups^a^

	Risks of parental mental disorders (OR, 95% CI; p-value)											
	Schizophrenia		Bipolar disorder		Depressive disorder		Alcohol use disorder		Substance use disorder		OCD	
Control group	1 (ref.)		1 (ref.)		1 (ref.)		1 (ref.)		1 (ref.)		1 (ref.)	
	Total sample											
Schizophrenia only group	**5.77 (5.38–6.18)**	** < 0.001**	**3.32 (3.05–3.61)**	** < 0.001**	**2.02 (1.92–2.13)**	** < 0.001**	**1.69 (1.53–1.87)**	** < 0.001**	**1.67 (1.49–1.87)**	** < 0.001**	**1.76 (1.41–2.19)**	** < 0.001**
OCD only group	**1.54 (1.28–1.86)**	** < 0.001**	**2.41 (2.09–2.78)**	** < 0.001**	**2.57 (2.39–2.77)**	** < 0.001**	**1.72 (1.49–1.99)**	** < 0.001**	**1.91 (1.63–2.34)**	** < 0.001**	**7.79 (6.57–9.24)**	** < 0.001**
Dual diagnosis group	**4.36 (3.42–5.55)**	** < 0.001**	**3.55 (2.75–4.59)**	** < 0.001**	**2.73 (2.34–3.18)**	** < 0.001**	**1.62 (1.16–2.25)**	**0.005**	**1.99 (1.41–2.80)**	** < 0.001**	**7.58 (5.30–10.84)**	** < 0.001**
	Male sample											
Schizophrenia only group	**5.44 (4.95–5.99)**	** < 0.001**	**3.14 (2.80–3.53)**	** < 0.001**	**2.09 (1.95–2.24)**	** < 0.001**	**1.86 (1.63–2.12)**	** < 0.001**	**1.72 (1.48–2.00)**	** < 0.001**	**1.92 (1.43–2.58)**	** < 0.001**
OCD only group	**1.67 (1.31–2.13)**	** < 0.001**	**2.36 (1.95–2.86)**	** < 0.001**	**2.53 (2.29–2.80)**	** < 0.001**	**1.71 (1.40–2.08)**	** < 0.001**	**1.56 (1.24–1.97)**	** < 0.001**	**7.55 (5.96–9.55)**	** < 0.001**
Dual diagnosis group	**4.31 (3.19–5.83)**	** < 0.001**	**3.72 (2.72–5.08)**	** < 0.001**	**2.91 (2.43–3.51)**	** < 0.001**	**1.79 (1.20–2.67)**	**0.004**	**2.30 (1.54–3.43)**	** < 0.001**	**9.29 (6.12–14.09)**	** < 0.001**
	Female sample											
Schizophrenia only group	**6.18 (5.58–6.85)**	** < 0.001**	**3.53 (3.12–4.00)**	** < 0.001**	**1.94 (1.79–2.10)**	** < 0.001**	**1.51 (1.30–1.76)**	** < 0.001**	**1.60 (1.35–1.90)**	** < 0.001**	**1.58 (1.13–2.21)**	** < 0.001**
OCD only group	**1.37 (1.02–1.85)**	**0.038**	**2.49 (2.01–3.08)**	** < 0.001**	**2.61 (2.34–2.92)**	** < 0.001**	**1.73 (1.39–2.14)**	** < 0.001**	**2.36 (1.91–2.93)**	** < 0.001**	**8.09 (6.31–10.36)**	** < 0.001**
Dual diagnosis group	**4.39 (2.39–6.58)**	** < 0.001**	**3.23 (2.06–5.06)**	** < 0.001**	**2.42 (1.85–3.17)**	** < 0.001**	1.32 (0.73–2.40)	0.365	1.44 (0.74–2.79)	0.278	**4.92 (2.43–9.97)**	** < 0.001**
	Adolescent sample											
Schizophrenia only group	**6.47 (5.44–7.69)**	** < 0.001**	**3.70 (3.04–4.50)**	** < 0.001**	**2.29 (2.00–2.61)**	** < 0.001**	**1.59 (1.28–1.98)**	** < 0.001**	**2.18 (1.74–2.71)**	** < 0.001**	**2.04 (1.25–3.34)**	**0.004**
OCD only group	**1.63 (1.12–2.36)**	**0.010**	**2.63 (2.03–3.42)**	** < 0.001**	**2.74 (2.38–3.17)**	** < 0.001**	**1.45 (1.09–1.92)**	**0.010**	**1.86 (1.41–2.45)**	** < 0.001**	**8.67 (6.33–11.89)**	** < 0.001**
Dual diagnosis group	**6.56 (4.31–9.97)**	** < 0.001**	**4.70 (3.00–7.35)**	** < 0.001**	**3.07 (2.27–4.17)**	** < 0.001**	**2.04 (1.19–3.50)**	**0.010**	**2.02 (1.07–3.80)**	**0.030**	**6.70 (3.26–13.76)**	** < 0.001**
	Adult sample											
Schizophrenia only group	**5.59 (5.18–6.03)**	** < 0.001**	**3.23 (2.95–3.55)**	** < 0.001**	**1.97 (1.86–2.09)**	** < 0.001**	**1.71 (1.53–1.91)**	** < 0.001**	**1.54 (1.35–1.75)**	** < 0.001**	**1.68 (1.31–2.16)**	** < 0.001**
OCD only group	**1.55 (1.24–1.92)**	** < 0.001**	**2.35 (1.98–2.78)**	** < 0.001**	**2.52 (2.31–2.74)**	** < 0.001**	**1.86 (1.57–2.20)**	** < 0.001**	**1.94 (1.60–2.35)**	** < 0.001**	**7.55 (6.15–9.26)**	** < 0.001**
Dual diagnosis group	**3.66 (2.71–4.93)**	** < 0.001**	**3.18 (2.32–4.35)**	** < 0.001**	**2.64 (2.21–3.16)**	** < 0.001**	1.41 (0.92–2.15)	0.115	**2.02 (1.35–3.04)**	**0.001**	**7.83 (5.19–11.83)**	** < 0.001**

Bold type indicates *p*-value < 0.05

*OCD* obsessive–compulsive disorder, *OR* odds ratio, *CI* confidence interval

^a^adjusting for demographic characteristics (age, sex, residence, and income) and parental ages at offspring birth

When considering the impact of first onset of OCD vs. schizophrenia in the probands, the associations of the six mental disorders in the parents were different in the three dual-diagnosis subgroups (Table 3). The simultaneously diagnosed and the OCD-first diagnosed groups did not show any association with increased odds of alcohol use disorder in their parents than the matched controls, and the schizophrenia-first diagnosed group did not show an association with an increased odds of substance use disorder. Compared with the controls, the simultaneously diagnosed group had the highest odds for OCD (6.19, 3.92–9.77) in their parents, followed by schizophrenia (4.50, 3.42–5.91) and bipolar disorder (3.62, 2.70–4.86); the schizophrenia-first diagnosed group had the highest odds for OCD (5.49, 3.17–9.52) in their parents, followed by schizophrenia (4.09, 2.09–5.59) and bipolar disorder (3.96, 2.89–5.43); the OCD-first diagnosed group had the highest odds for OCD (6.45, 3.33–12.49) in their parents, followed by schizophrenia (3.18, 1.96–5.16) and depressive disorder (2.78, 2.14–3.61). Based on stratification by sex, we found that the associations between parental mental disorders and the sequence of diagnosis in the dual-diagnosis group were similar for the male probands. However, female probands had no association with alcohol use disorder and substance use disorder in their parents. Besides, female probands who were had OCD diagnosed first only showed an association with an increased odds of depressive disorder but not the other five mental disorders (2.27, 1.40–3.66). Notably, both male and female probands with OCD first, schizophrenia first, or simultaneous onset had more associations with increased odds of depressive disorder (range of OR 2.27–3.04).

**Table 3:** Risks of parental mental disorders between dual-diagnosis and control groups^a^

	Risks of parental mental disorders (OR, 95% CI; p-value)											
	Schizophrenia		Bipolar disorder		Depressive disorder		Alcohol use disorder		Substance use disorder		OCD	
Control group	1 (ref.)		1 (ref.)		1 (ref.)		1 (ref.)		1 (ref.)		1 (ref.)	
Dual diagnosis group												
Total sample												
Simultaneously	**4.47 (3.12–6.39)**	** < 0.001**	**3.66 (2.28–5.89)**	** < 0.001**	**2.86 (2.28–3.60)**	** < 0.001**	1.39 (0.82–2.37)	0.223	**1.94 (1.16–3.25)**	**0.011**	**7.98 (4.76–13.38)**	** < 0.001**
Schizophrenia first	**4.52 (3.04–6.72)**	** < 0.001**	**4.26 (2.58–7.05)**	** < 0.001**	**2.19 (1.65–2.91)**	** < 0.001**	**2.35 (1.45–3.81)**	**0.001**	1.77 (0.94–3.31)	0.075	**5.57 (2.77–11.23)**	** < 0.001**
OCD first	**3.96 (2.27–6.91)**	** < 0.001**	**3.12 (1.54–6.33)**	**0.002**	**3.43 (2.52–4.68)**	** < 0.001**	1.02 (0.42–2.47)	0.962	**2.45 (1.26–4.76)**	**0.008**	**10.08 (5.18–19.64)**	** < 0.001**
Male sample												
Simultaneously	**4.54 (2.92–7.08)**	** < 0.001**	**3.66 (2.28–5.89)**	** < 0.001**	**3.01 (2.27–4.01)**	** < 0.001**	1.44 (0.74–2.19)	0.281	**2.29 (1.26–4.18)**	**0.007**	**9.68 (5.67–17.76)**	** < 0.001**
Schizophrenia first	**4.05 (2.40–6.82)**	** < 0.001**	**4.26 (2.58–7.05)**	** < 0.001**	**2.28 (1.60–3.24)**	** < 0.001**	**3.03 (1.74–5.29)**	** < 0.001**	2.03 (0.95–4.29)	0.065	**5.78 (2.38–14.05)**	** < 0.001**
OCD first	**4.43 (2.34–8.37)**	** < 0.001**	**3.12 (1.54–6.33)**	**0.002**	**3.75 (2.61–5.40)**	** < 0.001**	0.88 (0.28–2.74)	0.819	**2.74 (1.29–5.82)**	**0.009**	**13.34 (6.54–27.21)**	** < 0.001**
Female sample												
Simultaneously	**4.32 (2.36–7.94)**	** < 0.001**	**3.18 (1.63–6.19)**	**0.001**	**2.62 (1.78–3.84)**	** < 0.001**	1.30 (0.54–3.16)	0.560	1.37 (0.51–3.69)	0.533	**5.39 (2.00–14.56)**	**0.001**
Schizophrenia first	**5.25 (2.85–9.66)**	** < 0.001**	**3.12 (1.47–6.64)**	**0.003**	**2.05 (1.28–3.29)**	**0.003**	1.35 (0.50–3.64)	0.554	1.36 (0.43–4.25)	0.600	**5.24 (1.67–16.44)**	**0.005**
OCD first	2.91 (0.91–9.21)	0.069	**3.59 (1.32–9.77)**	**0.012**	**2.74 (1.51–4.99)**	**0.001**	1.34 (0.32–5.44)	0.683	0.81 (0.44–7.34)	0.409	3.37 (0.47–24.21)	0.227
Adolescent sample												
Simultaneously	**7.62 (3.90–14.87)**	** < 0.001**	**3.97 (1.73–9.11)**	**0.001**	**2.75 (1.73–4.39))**	** < 0.001**	1.57 (0.64–3.83)	0.324	1.71 (0.63–4.62)	0.293	**9.64 (3.90–23.85)**	** < 0.001**
Schizophrenia first	**3.89 (1.21–12.57)**	**0.023**	**7.63 (3.62–17.85)**	** < 0.001**	**3.16 (1.77–5.65)**	** < 0.001**	**4.03 (1.86–8.72)**	** < 0.001**	**3.28 (1.20–8.97)**	**0.021**	**6.72 (1.63–27.60)**	**0.008**
OCD first	**4.12 (1.28–13.25)**	**0.017**	1.11 (0.15–8.00)	0.919	**3.59 (2.08–6.19)**	** < 0.001**	1.05 (0.26–4.25)	0.949	1.43 (0.35–5.82)	0.618	3.18 (0.44–22.97)	0.251
Adult sample												
Simultaneously	**3.31 (1.80–6.06)**	** < 0.001**	**3.53 (1.98–6.29)**	** < 0.001**	**2.92 (2.24–3.79)**	** < 0.001**	1.28 (0.66–2.48)	0.461	**2.09 (1.15–3.08)**	**0.016**	**7.37 (3.92–13.86)**	** < 0.001**
Schizophrenia first	**4.04 (2.25–7.24)**	** < 0.001**	**3.38 (1.79–6.37)**	** < 0.001**	**2.01 (1.45–2.78)**	** < 0.001**	1.79 (0.95–3.35)	0.070	1.38 (0.61–3.08)	0.438	**5.27 (2.35–11.85)**	** < 0.001**
OCD first	**4.67 (2.18–10.00)**	** < 0.001**	**4.34 (2.03–9.27)**	** < 0.001**	**3.41 (2.34–4.97)**	** < 0.001**	1.00 (0.32–3.12)	0.996	**3.17 (1.49–6.74)**	**0.003**	**13.99 (6.99–28.48)**	** < 0.001**

Bold type indicates *p*-value < 0.05

*OCD* obsessive–compulsive disorder, *OR* odds ratio, *CI* confidence interval

^a^Adjusting for demographic characteristics (age, sex, residence, and income) and parental ages at offspring birth

## Discussion

In this national family-link cohort study, we found that all the three groups (OCD, schizophrenia, and dual diagnosis) showed increased odds of the six metal disorders in their parents, including schizophrenia, bipolar disorder, depressive disorder, substance use disorder, alcohol use disorder, and OCD. When considering the sequence of diagnoses in the dual-diagnosis group, irrespective of whether probands had first onset of schizophrenia, first onset of OCD, or simultaneous onset of both disorders, the odds for schizophrenia, bipolar disorder, depressive disorder, and OCD were still increased in their parents than those seen in the matched controls. However, compared with the control, the schizophrenia first-onset group had increased odds of parental alcohol use disorder, but it had no association with parental substance use disorder; on the other hand, the OCD first-onset group and the simultaneous-onset group had increased odds of parental substance use disorder, but it was not associated with parental alcohol use disorder. In addition, female probands with dual diagnosis had no association with increased odds of alcohol use disorder and substance use disorder than the matched controls. Besides, the female probands with dual diagnosis who had first onset of OCD only had an increased odds of depressive disorder.

To the best of our knowledge, the current study is the first cohort study to report the bidirectional relationship that probands with schizophrenia have higher parental odds of OCD while those with OCD have higher parental odds of schizophrenia. Multiple genetic liabilities for schizophrenia and OCD might explain the study findings. Schizophrenia and OCD share several gene polymorphisms (e.g., CACNA1C, COMT, and 5-HTTLPR), but schizophrenia still possesses other genetic polymorphisms distinct from those seen in OCD [16, 26, 27]. We also showed significantly increased odds of schizophrenia, bipolar disorder, depressive disorders, substance use disorders, alcohol use disorder, and OCD in the parents of probands with dual diagnosis than in the control probands. A previous cohort study by Poyurovsky et al. reported significantly higher odds of schizophrenia and OCD among the first relatives of probands with dual diagnosis compared with the control probands [4]. However, Poyurovsky et al. did not find increased odds for bipolar disorder, major depressive disorder, and substance use disorders in the parents of probands with dual diagnosis [4]. A genetic architecture study determined the top ten genes commonly associated with schizophrenia, OCD, and bipolar disorder, and these genes were implicated in the serotoninergic and dopaminergic synapses, calcium and cAMP pathways, and several addictive conditions, including alcohol, cocaine, and, amphetamine [8].

Our study showed increased parental odds of schizophrenia, bipolar disorder, OCD, substance/alcohol use disorder and depressive disorder in both probands diagnosed with OCD only and schizophrenia only group, consistent with the results of several previous family studies [4, 13, 15, 28–31]. Few studies have investigated parental odds of alcohol and substance use disorders among probands with OCD or schizophrenia. To date, only one small OCD family study (n = 153 probands) had reported negative results of the association between OCD and alcohol/substance disorder [32]. Addictive disorders and OCD share the characteristics of compulsive behaviors and are linked to dysfunction of the brain reward system [33]. Patients with OCD and substance/alcohol dependence display attenuated reward anticipation signals in the ventral striatum compared to those seen in the controls [33]. Low levels of dopamine D2-like receptors are related to both craving response in addicted patients and compulsions in patients with OCD [34, 35]. The serotonin-transporter-linked promoter region (5-HTTLPR) polymorphism is the best-supported variant for OCD, and it has been associated with alcohol and substance use disorders [36, 37]. Regarding schizophrenia, A prospective cohort study demonstrated that maternal cannabis use during pregnancy was associated with higher incidence of psychotic-like experiences in the offspring than in the controls [38]. The bidirectional relationship between substance use disorder and schizophrenia may imply the association of parental substance use disorder with increased risk of substance use disorder and schizophrenia in the offspring [39, 40]. A functional polymorphism in the catechol-O methyltransferase (*COMT*) gene was associated with reduced dopamine function in the prefrontal cortex and moderated the influence of substance use, and the *COMT* gene has been suggested in the pathogenesis of schizophrenia [41]. In a recent study analyzing a genetic database, common genes between OCD and schizophrenia were investigated [42]. The study reported that the most important genes associated with these disorders were SLC6A4, SLC6A3, and HTR2A, which are linked to the serotonergic synapse; DRD2 and COMT, associated with the dopaminergic synapse; GAD1 and DLG4, associated with the glutamatergic synapse; and BDNF and TH, associated with substance addiction. All these genes were found to be associated with the six mental disorders we investigated, thereby supporting our findings.

When considering the sequence of diagnosis and sex in the dual-diagnosis group, we could not find increased odds of parental schizophrenia, bipolar disorder, OCD, and substance use disorder in the females in the OCD-first group, when stratified by sex; however, the odds of these four mental disorders were increased among all probands of the OCD-first group. These associations might be explained by the lower proportion of females in the dual-diagnosis group (36.9%). For the same reason, the female schizophrenia-first group was not associated with an increased odds of parental alcohol use disorder. Importantly, when stratified by sex and by sequence of diagnosis, we found that all the subgroups showed associations with a higher odds of parental depression than the control group. The psychological stress that children with comorbid schizophrenia and OCD with poor cognitive and global function impose on their parents might be another factor associated with parental depressive disorder, besides genetic factors [1, 5].

In Taiwan [43], the community mental health care system has been established to provide case management for individuals with severe mental disorders, including schizophrenia, delusional disorder, bipolar I disorder, substance use disorder, alcohol use disorder, and organic brain disease with behavioral disturbance. As part of case management, assessments are conducted for the families of these individuals. Specifically, if there are more than one severe mental disorder patient within a single family, it is considered one of the criteria for identifying a vulnerable or fragile family. If the criteria for a vulnerable family are met, further interventions and family management are implemented. Our findings provide compelling evidence to support the implementation of such a systematic/familial mental healthcare policy by the government.

There are some limitations to the current study. First, the prevalence of major mental disorders, including OCD and schizophrenia, might be underestimated. Our study used data from the NHIRD, which is based on registration files and claims data; therefore, only the data of those patients who visited clinics or hospitals would be recorded in the database. Second, because of the relatively short study duration, we might have missed some probands with late-onset schizophrenia whose timing of disease onset was beyond the study period, thus limiting the power of the study. Third, familiality does not directly equate to genetic heritability. Other factors like environmental factors, life styles, or stress would be shared in families. These factors could be associated with the development of mental disorders; however, data on these are not available in the NHIRD.

In conclusion, in the current nationwide family-link cohort database, we found that OCD and schizophrenia were etiologically related to each other and that they were further related to bipolar disorder, depressive disorder, alcohol use disorder, and substance use disorder. Our findings could be used for genetic counseling and early awareness and intervention strategies for major mental disorders.

## Supplementary Information

Below is the link to the electronic supplementary material.

Supplementary file1 (DOCX 13 KB)

## Data Availability

As participants did not consent to the option of having their data publicly shared, or even anonymized, data will be made available to only potential collaborators with ethical approval after they submit a research proposal to Bureau of the NHI (https://nhird.nhri.org.tw/).
