# Supplementary material for: Parental mental disorders in patients with comorbid schizophrenia and obsessive–compulsive disorder: a nationwide family-link study
Source: Eur Child Adolesc Psychiatry. 2024 May 30;33(12):4325–34. doi: 10.1007/s00787-024-02480-0 (PMC11618191; doi:10.1007/s00787-024-02480-0)
Supplement: Supplementary file 1 — Supplementary file1 (DOCX 13 KB) [file 787_2024_2480_MOESM1_ESM.docx]

Supplementary material 1. SAS matching codes for case and controls

**proc** **sql**;

create table SCZOCD.control_pro2 as

select one.ID as study_id, two.ID as control_id,

one.id_birthday as study_birthday, two.id_birthday as control_birthday,

RAND('uniform') as r

from SCZOCD.study_case as one inner join SCZOCD.control_pro as two

on abs (one.ID_Birthday-two.ID_Birthday) <=**365**

and one.id_sex=two.id_sex

and one.urbanization=two.urbanization

and one.income=two.income;

**quit**;

**proc** **sql**;

create table SCZOCD.control_pro2_s as

select x.*

from SCZOCD.control_pro2 as x inner join (select control_id, min(r) as r from SCZOCD.control_pro2 group by control_id) as y

on x.control_id=y.control_id and x.r=y.r

order by x.study_id, y.r;

**quit**;

**data** control_id not;

set SCZOCD.control_pro2_s;

by study_id r;

retain num;

if first.study_id then num=**1**;

if num le **10** then do;

output control_id;

num=num+**1**;

end;

if last.study_id then do;

if num le **10** then output not;

end;

rename control_id=id;

keep control_id study_id num;

**run**;
